# Cell context-dependent dual effects of EFEMP1 stabilizes subpopulation equilibrium in responding to changes of *in vivo* growth environment

**DOI:** 10.18632/oncotarget.5220

**Published:** 2015-08-19

**Authors:** Yuanjie Hu, Chao Ke, Ning Ru, Yumay Chen, Liping Yu, Eric R. Siegel, Mark E. Linskey, Ping Wang, Yi-Hong Zhou

**Affiliations:** ^1^ Department of Biological Chemistry, University of California Irvine, Irvine, CA, USA; ^2^ Department of Surgery, University of California Irvine, Irvine, CA, USA; ^3^ State Key Laboratory of Oncology in South China and Collaborative Innovation Center for Cancer Medicine, Sun Yat-Sen University Cancer Center, Guangzhou, China; ^4^ UC Irvine Diabetes Center and Department of Medicine, University of California Irvine, Irvine, CA, USA; ^5^ Ziren Research LLC, Irvine, CA, USA; ^6^ Department of Biostatistics, University of Arkansas for Medical Sciences, Little Rock, AR, USA

**Keywords:** EFEMP1, glioma, tumor cell subpopulations, cell invasiveness, mitochondria respiration, tumor subpopulation equilibrium

## Abstract

Conflicting functions of EFEMP1 in cancer have been reported. Using two syngeneic glioma cell lines (U251 and U251-NS) carrying two different principal cell subpopulations that express high or low EGFR, and that are able to interconvert via mis-segregation of chromosome 7 (Chr7), we studied EFEMP1's cell-context-dependent functions in regulating subpopulation equilibrium, here defined by the percentage of cells carrying different copies of Chr7. We found that EFEMP1 attenuated levels of EGFR and cellular respiration in high-EGFR-expressing cells, but increased levels of NOTCH1, MMP2, cell invasiveness, and both oxidative phosphorylation and glycolytic respiration in low-EGFR-expressing cells. Consistently, EFEMP1 suppressed intracranial xenograft formation in U251 and promoted its formation in U251-NS. Interestingly, subpopulation equilibria in xenografts of U251-NS without EFEMP1 overexpression were responsive to inoculum size (1, 10 and 100 thousand cells), which may change the tumor-onset environment. It was not observed in xenografts of U251-NS with EFEMP1 overexpression. The anti-EGFR function of EFEMP1 suppressed acceleration of growth of U251-NS, but not the subpopulation equilibrium, when serially passed under a different (serum-containing adherent) culture condition. Overall, the data suggest that the orthotopic environment of the brain tumor supports EFEMP1 in carrying out both its anti-EGFR and pro-invasive/cancer stem cell-transforming functions in the two glioma cell subpopulations during formation of a single tumor, where EFEMP1 stabilizes the subpopulation equilibrium in response to alterations of the growth environment. This finding implies that EFEMP1 may restrain cancer plasticity in coping with ever-changing tumor microenvironments and/or therapeutic-intervention stresses.

## INTRODUCTION

Cell heterogeneity within a given tumor has long been observed in most cancers. Recently, it has been recognized as a cause of tumor resistance to treatment. Glioblastoma multiforme (GBM, WHO grade IV glioma) is morphologically, genetically, and cytogenetically heterogeneous, and uniformly fatal due its rapid cellular proliferation and strongly invasive behavior. One of the commonly found tumor-cell subpopulations in GBM has an abnormality in EGFR (overexpression, gene amplification, and/or mutation). EGFR abnormality is also one of the molecular hallmarks of GBM [1, 2]. Studies over the past decade have revealed the existence of an invasive subpopulation of cells pivotal to the initiation and re-colonization of GBM. These cells have variously been named as tumor stem cells, stem-like tumor-initiating cells of glioma, or glioma stem cells, based on shared molecular hallmarks and growth behaviors with neural stem cells. Enriching and maintaining this type of glioma cell in culture has been possible by using conditions adopted for glia precursor/neural stem cells [3-5]. Thus far, however, there is little understanding about how the equilibrium of cell types in tumor heterogeneity is regulated.

EFEMP1, a fibulin-like, extracellular protein, was initially identified as a senescence protein [6, 7]. Then its function in the eye was discovered after finding a germline mutation of *EFEMP1* in retinal dystrophy [8]. During the past decade, numerous reports have revealed EFEMP1 as an important player in cancer initiation and progression, by both tumor-suppressive and oncogenic effects in different cancer-cell contexts [9]. Although the anti-angiogenic [10] and anti-EGFR [11] effects of EFEMP1 may account for its anti-tumor effect in most cancer types, EFEMP1's tumor-promoting role has been related to activation of NOTCH signaling [12], and hence is likely associated with conditions in the stem-like cancer-cell context [13, 14]. In a previous study, we demonstrated, in glioma cell line U251, the co-existence of two cell subpopulations that were experimentally determined to be tumor mass-forming cells (TMC) and stem-like tumor-initiating cells (STIC). Through mis-segregation of chromosome 7 (Chr7), the two types of cell interconvert to reach an equilibrium optimal for overall tumor growth *in vitro* and *in vivo* [15]. Because TMC and STIC of U251 differentially expressed EGFR and NOTCH1, we carried out a study on the impact of EFEMP1 on tumor heterogeneity, based on its potential twofold effect in these two different cell subpopulations. In this case, the cell types were distinguished by their carrying a different copies of Chr7. Here, we show that such conflicting roles of EFEMP1 in cancer occurrs in two cell subpopulations forming one tumor, and for the first time show EFEMP1's contradictory effect in the regulation of cellular respiration in the two cell subpopulations. Together, these findings may explain the observed ability of EFEMP1 to stabilize subpopulation equilibrium in an orthotropic xenograft model in responding to changes in the tumor microenvironment.

## RESULTS

### Context-dependent function of EFEMP1 on EGFR and NOTCH signaling pathways, cell invasiveness, and respiration profile of two tumor-cell subpopulations

**Figure 1:**
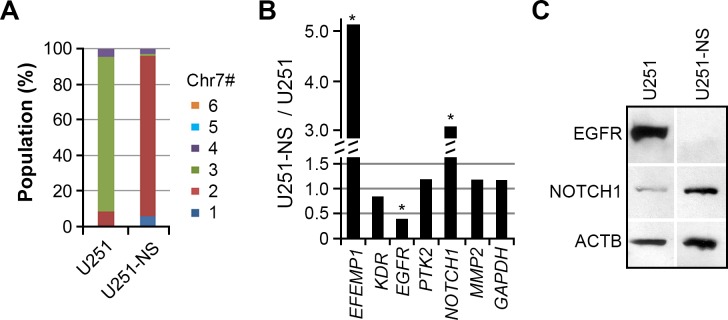
Differential expression of EGFR and NOTCH1 in TMC and STIC subpopulations of U251 **A.** Differential proportions of cell subpopulations with copy number variation (CNV) for chromosome 7 (Chr7) in parental (U251) and clonal neural sphere subculture of U251 (U251-NS). The proportion was calculated based on more than 250 cells subjected to FISH analysis using dual probes for EGFR and CEP7. **B.** Differential gene expression between U251 and U251-NS, normalized to *ACTB*. Each gene was quantified by AqRT-PCR in 2-4 repeats to show less than 20% variation. Genes differently expressed at a level > 2 fold are labeled. **C.** Validation of different expression levels of EGFR and NOTCH1 by immunoblotting.

In a prior study, we have shown intratumoral heterogeneity in all analyzed glioma specimens, with tumor-cell subpopulations carrying different copies of Chr7 [15]. The principal subpopulations of glioma cell line U251 and its derived neural sphere subculture line (U251-NS) respectively carried three and two copies of Chr7 (Figure 1A) and were experimentally determined as TMC and STIC [15]. Comparison of gene expressions, normalized by *ACTB*, showed higher expression of *EFEMP1* and *NOTCH1*, and lower expression of *EGFR* in U251-NS compared to U251 (Figure 1B). The oppositely directed expression patterns of *EGFR* and *NOTCH1* in these two syngeneic glioma cell lines were confirmed at the protein level by immunoblotting (Figure 1C). In contrast, there were no marked differences in the expressions of genes encoding other tyrosine kinases (*KDR*, *PTK2*), the matrix metallopeptidase gene *MMP2*, or the internal reference gene *GAPDH*.

**Figure 2:**
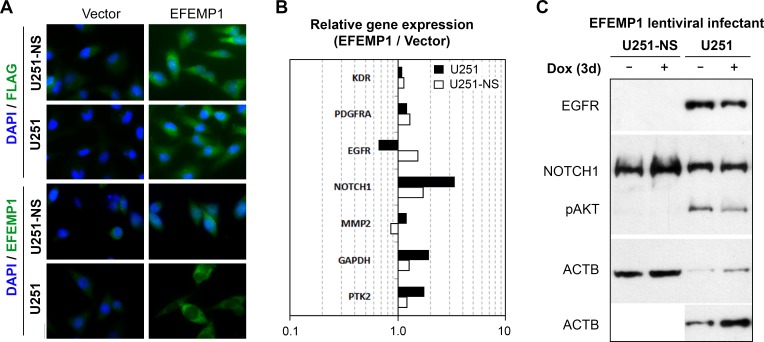
Cell context-dependent regulation of gene expressions by EFEMP1 **A.** Immunofluorescence from antibodies for FLAG and EFEMP1 for ectopic EFEMP1 carrying an *N*-terminal FLAG tag and endogenous/ectopic EFEMP1 expressed by lentiviral infected cells. **B.** Comparison on the effect of EFEMP1 on the expression of relevant genes, normalized to *ACTB*, in U251 and U251-NS. **C.** EGFR and NOTCH1 expression by immunoblotting. Cells were cultured in medium containing doxycyclin (Dox, 1 ug/ml) for 3 days prior analyses.

We previously showed that overexpression of EFEMP1 nearly abolished the tumorigenicity of U251 in both subcutaneous and intracranial models due to its anti-angiogenesis and anti-EGFR functions [11, 16]. To determine the effect of EFEMP1 in U251-NS, we transduced the cells using lentiviral vectors, either pTRIPZ (vector alone) or pTRIPZ-EFEMP1 (with or without an N-terminal FLAG tag), the same as was done previously for U251 [11]. The transgene expression was verified by immunocytofluorescence (Figure 2A) and real-time qRT-PCR. U251-NS, which has a five-fold higher level of endogenous *EFEMP1* transcript than U251 (Figure 1B), showed only a modest 2-fold increase in transcript levels after transfection, much smaller than the 20-fold increase exhibited by U251 (data not shown).

We have shown a reduction of membrane EGFR in response to a 3-day induction of EFEMP1 in U251 [11]. Here, we further showed in Figure 2B a 1.5-fold decrease of *EGFR* transcripts by EFEMP1 overexpression in U251, which was consistent with the immunoblotting results shown in Figure 2C. In contrast, a 1.5-fold increase of *EGFR* transcripts was seen in U251-NS, but could not be confirmed by immunoblotting due to the absence of detectable EGFR in the whole-cell lysate. In both U251 and U251-NS, *NOTCH1* gene expression was upregulated (3.4 and 1.7-fold in U251 and U251-NS, respectively), but an upregulated NOTCH1 protein expression was only seen in U251-NS. For comparison, we examined the effect of EFEMP1 on three other genes (*MMP2*, *KDR*, and *PDGFPA*) and found for all three a change of less than 1.5-fold in both lines. There was a 1.7-fold increase of *PTK2* and 1.9-fold increase of *GAPDH* in U251, which were not verified by immunoblotting.

**Figure 3:**
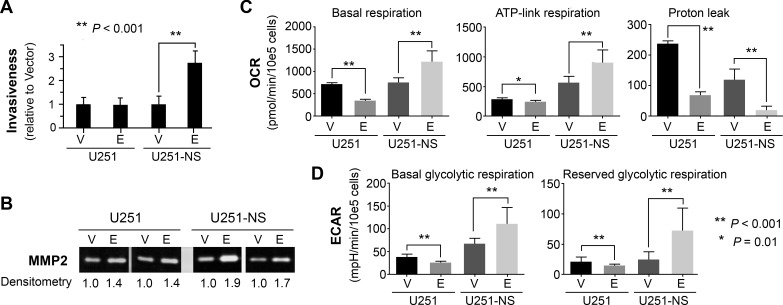
STIC-context-specific effect of EFEMP1 on increasing cell invasiveness via MMP2 activation and increasing metabolomics of cancer stem cell **A.** Comparison of EFEMP1's effect on cell invasion through Matrigel, with pTRIPZ-Empty (vector) infected cells set to unity. **B.** Two gelatin zymography assays showed the level of activated MMP2. **C.**-**D.** Comparison EFEMP1's effect on mitochondrial respiration. Oligomycin (a complex V inhibitor), FCCP (an uncoupler), and Rotenone (a complex I inhibitor) was injected sequentially to assay the mitochondrial respiration capacity. The basal respiration, ATP-linked respiration (oligomycin-sensitive fraction), proton leak (oligomycin-insensitive fraction), basal glycolytic respiration and reserved glycolytic respiration was compared and presented. Error bars correspond to the SD of triplicate measurements. OCR, basal oxygen consumption rate; ECAR, extracellular acidification rates.

The effect of EFEMP1 on cell invasiveness was examined by a Matrigel invasion assay. As shown in Figure 3A, EFEMP1 overexpression in U251 did not show any effect on cell invasiveness. In contrast, EFEMP1 significantly increased U251-NS invasiveness (2.7-fold). Glioma-cell invasiveness was often positively correlated with up-regulation of MMP2 or increase of MMP2 activation [17]. A gelatin zymography assay was performed using protein precipitated from conditioned media of cells cultured in basal medium for 48 hours. As shown in Figure 3B, overexpression of EFEMP1 in U251 slightly enhanced MMP2 activity (1.4-fold), while in U251-NS, it nearly doubled MMP2 activity. Based on the result of qRT-PCR, which did not show an effect of EFMEP1 on *MMP2* gene expression (Figure 2B), the observed EFEMP1-mediated up-regulation of MMP2 activity was likely from its enhancing the activation of MMP2 in the extracellular compartment.

The effect of EFEMP1 on cellular bioenergetics was examined using an extracellular flux analyzer. As shown in Figure 3C from measurements of oxygen consumption rate (OCR, as a measure of mitochondrial respiration), basal respiration in U251 was significantly attenuated by EFEMP1, due to the combined effects of a decrease in ATP-linked respiration and a proton leak. In contrast, basal respiration in U251-NS was significantly enhanced by EFEMP1 due to an increase of ATP-linked respiration, even though there was a decrease in the proton leak, which contributed about 15% of the basal respiration in vector-transduced U251-NS. As shown in Figure 3D, from measurements of extracellular acidification rate (ECAR, as a measure of glycolysis), basal glycolytic respiration and reserved glycolytic respiration (induced by Oligomycin) in U251 were both significantly decreased due to EFEMP1 overexpression, whereas in U251-NS both were significantly increased due to EFEMP1 overexpression. The reserve respiration capacity could not be detected in U251 and U251-NS with or without overexpression of EFEMP1 (not shown), suggesting that the mitochondrial respiratory capacity was exhausted in both TMC and STIC of U251.

### Reversal of effect of EFEMP1 in regulating tumorigenicity depending on initial cell inoculum size

**Figure 4:**
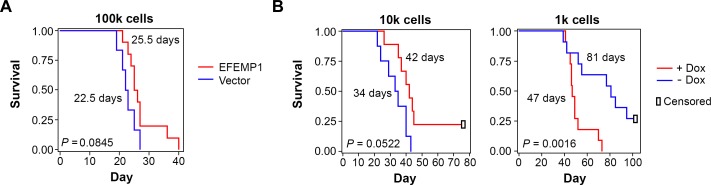
Kaplan-Meier survival curves of mice with i.c. implantation of U251-NS without or without overexpression of EFEMP1 **A.** EFEMP1 or Vector-transduced cells effect with inoculum size of 100,000. **B.** EFEMP1-transduced cells with or without Dox-induced EFEMP1 overexpression, with inoculum size of 10,100 and 1,000. The *P* values were from Log-Rank statistics on pairwise comparisons of the two groups via Cox Regression. Median survival days of mice post implantation of tumor cells were shown by the survival curve.

Because the study described above showed a pro-invasive effect of EFEMP1 in STIC-enriched U251-NS, we wondered if an oncogenic effect of EFEMP1 would be seen in orthotopic xenograft formation. We implanted 100,000 cells of lentiviral-vector transduced or lentiviral-EFEMP1-transduced U251-NS into the frontal lobes of nude mice (10 mice per group) and obtained survival data based on criteria described in Methods. To our surprise, as shown in Figure 4A, overall survival was not significantly shorter in mice implanted with U251-NS (EFEMP1), in comparison to that of U251-NS (Vector). On the contrary, there was a consistent improvement of 2-3 days in the 25^th^, 50^th^, and 75^th^ percentiles of survival by EFEMP1 overexpression in U251-NS, but it was not statistically significant (two-sided *P* = 0.085). We therefore hypothesized that the co-existence of 5% TMC in U251-NS, 5000 TMC when implanting 100,000 cells, might play a significant role in driving tumor onset by inducing angiogenesis, which was suppressed by the presence of EFEMP1.

We then performed i.c. implantation with 10,000 and 1,000 cells of U251-NS (EFEMP1) with or without doxycycline (Dox)-induced overexpression of EFEMP1, 1 microgram/ml for three days prior to implantation. Dox-induced EFEMP1 expression was maintained post-implantation by feeding mice with Dox-containing water (1 mg/ml) throughout the experiment. This experiment was performed twice (five mice per group in each experiment), and survival data were combined. The absence of significant treatment vs experiment interactions showed the validity of combining the data from the two independent experiments. In the i.c. xenograft model of U251-NS with an inoculum size of 10,000 cells, as shown in the left panel of Figure 4B, a similar survival difference was observed between the EFEMP1 and Vector groups as that from an inoculum size of 100,000 cells (Figure 4A). In contrast, the tumor-promoting effect of EFEMP1 was clearly shown in the i.c. xenograft model with inoculum size of 1,000 cells. As shown in the right panel of Figure 4B, there was a dramatic (*P* < 0.05) shortening of survival time as a result of Dox-induced EFEMP1 expression.

### EFEMP1 stabilizes cell-subpopulation equilibrium from changing in response to different *in vivo* growth environments

**Figure 5:**
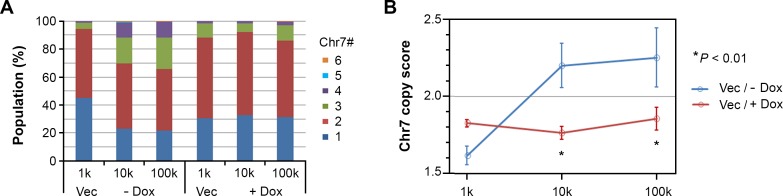
EFEMP1 suppressed the alteration of the cell subpopulation equilibrium *in vivo* **A.** Proportions of cell subpopulations in i.c. xenografts from different groups of mice shown in Figure 4. **B.** Comparison of Chr7 copy score (average Chr7 number per cell in xenografts from each group) for the effect of EFEMP1 induced by Dox. Error bar is SD of data from 2-3 xenografts.

We examined three i.c. xenografts of each group described in Figure 4 by EGFR/CEP7 FISH. Based on Chr7 counts in 250-300 cells per tumor, Chr7 number averages of each population, with Chr7 number ranging from 1 to 6, were plotted (Figure 5A). The counting result showed similar proportions of cell subpopulations in xenografts derived from implantation of both 10,000 and 100,000 cells of U251-NS when EFEMP1 was not overexpressed, but a substantial difference was found in xenografts from implantation of 1,000 cells, with an increase of cells having 1 copy of Chr7 and a decrease of cells with 3 copies of Chr7. In contrast, a similar equilibrium of Chr7 subpopulations was seen in xenografts of U251-NS when EFEMP1 was overexpressed for all three inocula. A Chr7 copy score was then computed for each i.c. xenograft, which was the average number of FISH-detected copies per cell, and plotted against inoculum size. As shown in Figure 5B, in absence of EFEMP1 overexpression, copy score was very responsive to inoculum size, while in presence of EFEMP1 overexpression, copy score was very un-responsive to inoculum size.

**Figure 6:**
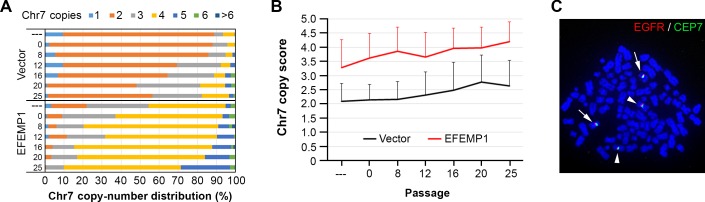
EFEMP1 does not suppress the alteration of subpopulation equilibrium *in vitro* **A.** Proportion of subpopulations in U251-NS before and after serial, three-day passages in serum adherent culture conditions, using the same cell plating density (5×10^5^/100 mm dish), with Chr7-cell types determined by FISH. **B.** Comparison of Chr7 copy score in overall cells over passages. Error bar was SD of data from number of cells examined. **C.** A metaphase picture of FISH showing near tetraploid nucleus with two copies of normal (*arrowhead*) and two copies of deleted (*arrow*) Chr7.

The effect of EFEMP in suppressing changes in subpopulation equilibrium in xenografts of U251-NS was also studied following a change of *in vitro* culture conditions. We previously have demonstrated reversal of cell subpopulation equilibrium of U251-NS towards U251, by changing the culture conditions of U251-NS (a non-adherent or fibronectin-anchored condition with medium supplemented with growth factors) to those used for U251 (a regular cell culture condition with medium supplemented with bovine serum) [15]. As shown in Figure 6A, there was a similar change of subpopulation equilibrium in lentiviral Vector-transduced U251-NS, with trends of an increase in the proportion of TMC and a decrease of STIC.

In contrast to EFEMP1's *in vivo* suppression of any alteration of subpopulation equilibrium when tumor onset conditions were changed (see Figure 5), EFEMP1-transduced U251-NS showed a similar increase of the Chr7 copy score as seen for that of empty-Vector-transduced cells in response to a change of culture conditions, EFEMP1 overexpression in U251-NS also led to maintaining a large proportion of near tetraploid cells in the culture, with a majority of cells carrying 4 copies of Chr7. Metaphase images of FISH verified that the majority of 4-Chr7 cells in EFEMP1-transduced U251-NS were derived from near diploid STIC, as they has two normal Chr7 and two Chr7 with a deletion, where diploid STIC have only one of each [15].

### EFEMP1 suppressed the changes of EGFR-driven growth status of STIC

**Figure 7:**
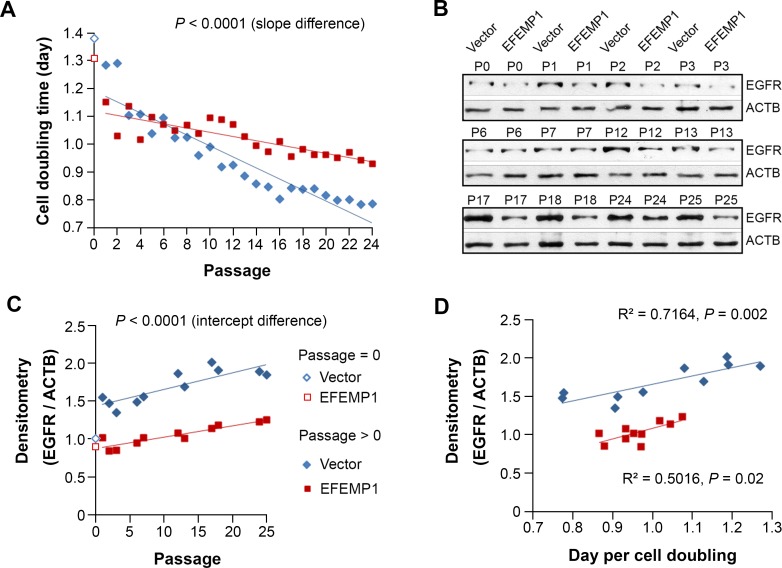
EFEMP1 suppressed the growth of STIC by suppressing the increase of EGFR Comparison between U251-NS (Vector) and U251-NS (EFEMP1) on cell doubling time **A.** and EGFR protein level **B.**-**C.** in cultures described in Figure 6. **D.**, Pearson correlation analysis on cell growth speed and EGFR level.

We have found that, after changing the culture environment of U251-NS by changing to the serum-containing adherent culture conditions from serum-free neural sphere culture conditions, the subpopulation equilibrium gradually reverted back to that of U251, along with an increase in the overall cell-growth rate [15]. We repeated this assay with U251-NS (Vector) or U251-NS (EFEMP1), with results of Chr7-FISH analysis described above. Here Figure 7A showed the change of cell doubling time over 24 passages. The increase in cell growth rate over serial passage was seen in both U251-NS (Vector) (blue diamonds) and U251-NS (EFEMP1) (red squares), but the speed of increase was significantly suppressed by overexpression of EFEMP1.

Testing whole cell lysate for EGFR by immunoblotting showed a rapid increase of EGFR in U251-NS (Vector) one passage after changing growth conditions, which continued over serial passages (Figure 7B). In contrast, EFEMP1 nearly blocked the increase of EGFR during passages 1-7. The subsequent increase of EGFR could be explained by an increasing number of tetraploid cells carrying 4 and 5 copies of Chr7 (Figure 6). Pearson correlation analysis showed significantly increasing levels of EGFR with cell passage number, both for U251-NS (Vector) (*R* = 0.845; *P* = 0.0011) and for U251-NS (EFEMP1) (*R* = 0.917; *P* < 0.0001) (Figure 7C). Further statistical analysis showed a significant increasing trend for correlation of EGFR with cell growth rate for U251-NS (Vector), but this was not as strikingly evident in U251-NS cells with overexpression of EFEMP1 (Figure 7D). These observations suggest a change in the role of EFEMP1 from pro-invasive to anti-proliferation in STIC where EGFR expression increased in response to a change in culture conditions, and EFEMP1 showed anti-EGFR function.

## DISCUSSION

Overall, our data, obtained by studying two cell subpopulations having different molecular contexts, with varying phenotypes designated as STIC and TMC, demonstrates the cell-context-dependent dual action of EFEMP1, by promoting MMP2 activation and invasion of STIC, while suppressing EGFR/pATK growth signaling and *in vivo* growth of TMC. For the first time, it showed EFEMP1's contradictory effect in regulation of cellular respiration. A respiration profile of higher oxidative phosphorylation (OXPHOS) was reported in neurospheres of a glioma cell line (U87) and a GBM primary culture (GBM146), in comparison to their monolayer cultures [18], and gliomaspheres were dependent on OXPHOS for their energy production [19]. In contrast, normal stem cells are more glycolytic than differentiated cells, and reprogramming induces a bioenergetic conversion from an oxidative to a glycolytic state [20]. All together, these suggest that in the STIC context, EFEMP1 has a cancer stem cell transforming effect via enhancing both oxidative phosphorylation and aerobic glycolysis, which is consistent with the *NOTCH1* up-regulation (Figure 2). EFEMP1's strong tumor-suppression effect in TMC was consistent with reduction in both oxidative phosphorylation and aerobic glycolysis (Figure 3).

In a previous study, we demonstrated the inherent nature of tumor heterogeneity in glioma, characterized by chromosome mis-segregation and a dynamic equilibrium of cell subpopulations [15]. Here, for the first time, EFEMP1 is shown to suppress the alteration of the tumor cell subpopulation equilibrium triggered in response to a chan­­ge of the orthotopic *in vivo* growth environment. Overall, the data suggest a role of EFEMP1 in checking cancer plasticity, which is hypothesized to be related to its cell-subpopulation context-dependent dual function.

In addition to its dual functions in the two key cell subpopulations in tumor formation, the effect of EFEMP1 on maintaining a relatively stable subpopulation equilibrium may include its role in suppressing mis-segregation of Chr7, which drives diversification of cell subpopulations. We have shown that a change in the growth environment or subjecting cells to radiation changes the equilibrium between cell subpopulations [15]. The dramatic increase in the proportion of cells having one Chr7 copy, up to 40% in xenografts derived from a small inoculum (1000 cells) of U251-NS, is a validation of this hypothesis. Increased diversification of cell populations is related to enhanced tumor plasticity and adaptability, but could retard overall tumor growth. The effect of EFEMP1 in promoting the i.c. tumorigenicity of U251-NS after implantation of 1000 cells may be due to its enhancing of MMP2-mediated invasion by STIC along with enhanced OXPHOS and glycolytic metabolism. Alternatively, the effect may be due to suppression of alteration of the cell subpopulation equilibrium.

Tumor heterogeneity is well recognized in cancer, with evidence supporting a link to cancer plasticity, therapeutic resistance, and subsequent tumor recurrence/progression. However, it is rarely taken into consideration in experimental cancer models for study of cancer biology and the development/testing of anti-cancer therapeutics. Here, we demonstrate different equilibria of subpopulation cells having different Chr7 numbers, in derived i.c. xenografts, in response to the implantation of different numbers of tumor cells. The Chr7 copy score increased along with the increase of inoculum size. Interestingly, the increase of Chr7 copy score was negatively correlated with survival of mice, which coincides with the observation in gliomas, that an increase of Chr7 copy score is positively associated with the grade of glioma [15].

In addition to intratumoral heterogeneity, different GBM subtypes have been identified based on molecular markers that predict outcome of patients undergoing chemotherapy and radiation therapies [2]. We demonstrated *EGFR*'s unfavorable prognostic values in a subtype of gliomas expressing a low level of *EFEMP1*, which was consistent with EFEMP1's anti-EGFR function. An unfavorable prognostic value of *EFEMP1* was also observed in a subtype of gliomas expressing a low level of *EGFR* [11]. Our data from this study support this clinical observation, by demonstrating a pro-invasive oncogenic effect of EFEMP1 in the STIC subpopulation not expressing EGFR.

Interestingly, the contradictory role of EFEMP1 in glioma prognosis was shown in the degrees of i.c. tumorigenicity of U251-NS associated with different inoculum size (100k, 10k, and 1k cells). In the three groups of xenografts from cells without EFEMP1 overexpression, we found changes in subpopulation equilibrium with an increase of the Chr7 copy score, as a function of inoculum size, which was also negatively correlated with mouse survival. In the three groups of xenografts from cells with EFEMP1 overexpression, the Chr7 copy score remained unchanged; however, mouse survival was either prolonged or shortened due to overexpression of EFEMP1. This seeming contradiction is apparently due to the combined effect of EFEMP1 suppression of TMC-driven tumor onset and growth, promoting STIC invasion and survival, and possibly blockage of chromosome mis-segregation of STIC in giving rise to slowly-proliferating cells with 1 copy of Chr7.

The data obtained in this study verified the effect of EFEMP1 in suppressing EGFR-driven cancer cell growth, and demonstrated a new activity of EFEMP1 in suppressing cell type/proportion change within a tumor, thereby maintaining existing intra-tumoral heterogeneity. All these findings are based on our ability to distinguish two functionally distinct cell subpopulations by their having different numbers of Chr7 and the finding of Chr7 mis-segregation in maintaining cell-subpopulation diversity. The finding that EFEMP1 is, overall, tumor-suppressive is consistent with the favorable effect of *EFEMP1* expression in the overall prognosis of patients with GBM [16], breast [21], liver [22], lung [23], and nasopharynx [23], and the finding that *EFEMP1* is downregulated and/or silenced via methylation of the *EFEMP1* promoter in cancer tissues of breast [21], colon [24], liver [22], lung [25, 26], nasopharynx [23], and prostate [27, 28], and recently in gastric cancer [29]. EFEMP1's transforming and pro-invasive functions may play a key role in cervical [30] and ovarian [31] carcinoma, where EFEMP1 expression was correlated with poor prognosis.

## MATERIALS AND METHODS

### Glioma cell lines and lentivirus transduction of EFEMP1

The human glioma cell line U251 was obtained from Dr. Alfred Yung's lab at the University of Texas M.D. Anderson Cancer Center. U251-NS was established in UC Irvine as described previously [15]. All cells were cultured in 37°C humidified CO_2_ (5%) incubators in DMEM/F12 medium supplemented either with 5% bovine serum in cell culture dishes (U251), or with growth factors (10 ng/ml of EGF and bFGF and 1% B27) in fibronectin (100 ng/ml) coated dishes (U251-NS). Construction of lentiviral vectors of EFEMP1 in pTRIPZ and production of lentivirus of Vector and EFEMP1 have been described previously [11].

### Immunocytofluorescence and immunoblotting

U251 and U251-NS cells transduced by pTRIPZ-Vector or EFEMP1 were grown in poly-lysine-coated chamber slides in each defined culture medium without or with doxycycline for 2 days (1ug/ml) prior to use for immunocytofluorescence detection of FLAG and EFEMP1. In brief, cells were rinsed with cold PBS, fixed with 4% PFA for 10min and blocked with 10% donkey serum for 0.5-1 hour at room temperature, then incubated with primary antibody (1:1000 blocking solution) overnight at 4°C. Alexa 488nm donkey anti-rabbit IgG (Invitrogen, Carlsbad, CA) was then applied for 1 hour at room temperature. Cells were then mounted in medium containing DAPI, and pictures were taken using a fluorescence microscope with a 60X lens. A standard immunoblotting protocol was followed to detect proteins in whole cell lysate, using antibodies against EGFR, NOTCH1, and ATCB obtained from Cell Signaling (Danvers, MA), diluted according to the manufacturer's recommendations.

### Intracranial (i.c.) xenograft models

For formation of i.c. xenografts, transduced glioma cells (1, 10, and 100 thousand of cells / 3 μl DMEM/F12) were injected 3 mm into the frontal lobe, at spots of 2.5 - 3 mm right of bregma and 1.8-2 mm anterior to coronal suture, of 4-to-6-week-old female nude mice (stain NCrNu-M, Taconic, Hudson, NY) using a stereotactic frame with micro manipulator, and with injection speed (1.5 μl/min) controlled by a Harvard Apparatus Model 11 Plus syringe pump. Mice were observed daily, weighted 1-3 times a week, until moribund signs (hunchback posture and/or 20% weight loss) appeared, and were terminated the following day (which was recorded as the survival date). Brains of nude mice were then dissected, fixed in 4% paraformaldehyde in 4°C for 1 day, dehydrated in 30% sucrose for 1 day, mounted for optical coherence tomography (OCT) freeze-drying on dry ice, and then sliced on a cryostat for histology and FISH analyses.

### Fluorescence *in situ* hybridization (FISH) and analysis of glioma cell heterogeneity with Chr7-CNV in i.c. xenografts

Cryosections (6 μm) of i.c. xenografts were fixed with 100% methanol for 5 minutes. The slides were further treated with in 0.3% sodium citrate solution for 10 minutes in a pressure cooker, and rinsed with water briefly before FISH analysis using Vysis LSI EGFR SpectrumOrange / CEP 7 SpectrumGreen Probes (Abbott Molecular Inc) following the manufacturer's instructions. Cells were counted on slides using a Nikon Eclipse TS100/TS100F fluorescent microscope with a 100X lens. The Chr7 copy score for each tumor was calculated as the average number of FISH-detected copies per cell among 250-300 cells counted.

### Gelatin zymography and matrigel invasion assays

Gelatin zymography and Matrigel invasion assays were performed following procedures described previously [17]. Briefly, proteins in 24 hour-conditioned cell culture medium (serum-free) were precipitated with 4 volumes of cold acetone, spun immediately at 14,000 rpm for 5 minutes at 4°C, and resuspended in radioimmunoprecipitation assay buffer (RIPA) containing Protease Inhibitor Cocktail (Roche). Equal amounts of conditioned medium protein were examined using gelatin zymography. 2-5×10^5^ cells in 200-500 μl serum-free medium were loaded in 2-3 replications on Matrigel (1 μg/ml)-coated trans-well plates (8μm; Fisher Scientific), with 0.5-1 ml medium containing 0.05% FBS. 48 hours later, cells were stained using the HEMA3 CMS kit (Fisher Sci). Images of invading cells were taken under a microscope using a 20X lens. Twenty images were taken per trans-well and cells were counted for statistical analysis.

### Real-time qRT-PCR

Total RNA (∼1 μg) was converted by into cDNA using 5 pmol oligo d(T)20VN and 5 units of Superscript III reverse transcriptase (Invitrogen) following the manufacturer's protocol. The cDNA samples were diluted and gene expressions were quantified using a real-time qRT-PCR (SYBR Green I) system that has a single standard incorporating both the marker and reference genes [32]. The primer sequences for genes in qRT-PCR are available from Ziren Research LLC (www.zirenresearch.com) upon request.

### Assessment of cellular bioenergetics through flux analysis

To measure respiration and mitochondrial function in giloma cells, we employed a Seahorse Bioscience XF24 Extracellular Flux Analyzer (Seahorse Bioscience, North Billerica, MA) and followed the manufacturer's protocol. Briefly, cells were seeded in a non-coated or fibronectin-coated 24-well Seahorse XF-24 assay plate at various initial plating densities according to each cell line's growth speed, with 2×10^5^ cells of U251 (Vector or EFEMP1) or 5×10^5^ cells of U251-NS (Vector or EFEMP1) per well before analysis. On the day of metabolic flux analysis, cells were washed once with unbuffered DMEM/F12 (pH7.4) and incubated at 37°C in the same media in a non-CO_2_ incubator for 1 hr. Four baseline measurements of OCR and ECAR were taken before sequential injection of the following mitochondrial inhibitors (final concentration): oligomycin (1 μg/ml), FCCP (0.3 μM) and rotenone (0.1 μM). Three measurements were taken after addition of each inhibitor. OCR and ECAR values were automatically calculated and recorded by the Seahorse XF-24 software. The basal respiration was calculated by averaging the four measurements of OCR before injection of inhibitors.

### Statistical analysis of data on intracranial xenografts

Overall survivals of mice bearing intracranial glioma xenografts were estimated via Kaplan-Meier survival curves, and compared for equality between EFEMP1-expression groups at each cell concentration via stratified log-rank tests in which the experiment (1^st^ or 2^nd^) was the stratification factor. The validity of stratifying on experiment was examined via Cox regression with group, experiment, and their interaction as factors, specifically by testing the significance of the group-x-experiment interaction. The effects of Dox (to induce EFEMP1) and inoculum size on Chr7-copy scores were determined via 2-way ANOVA, with post-hoc analysis concentrating on (a) the effect of inoculum size within each Dox group, and (b) the effect of Dox at each inoculum level. The significance level was set at *P* < 0.01 in order to adjust for the multiple comparisons without overinflating Type II error. SAS versions 9.2 and 9.3 (The SAS Institute, Cary, NC) were used for all analyses.

## Abbreviations

FISH: fluorescence in situ hybridization
OCR: oxygen consumption rate
ECAR: extracellular acidification rate
Chr7: chromosome 7
